# *N*-2-Hydroxypropyl Trimethyl Ammonium Chloride Chitosan as Adjuvant Enhances the Immunogenicity of a VP2 Subunit Vaccine against Porcine *Parvovirus* Infection in Sows

**DOI:** 10.3390/vaccines9091027

**Published:** 2021-09-16

**Authors:** Kai Zhao, Yuan Gao, Gaowei Hu, Lei Wang, Shangjin Cui, Zheng Jin

**Affiliations:** 1 Institute of Nanobiomaterials and Immunology, School of Life Science, Taizhou University, Taizhou 318000, China; zhaokai@tzc.edu.cn (K.Z.); gaoyuan@tzc.edu.cn (Y.G.); hugaowei68@163.com (G.H.); leiwang@chnu.edu.cn (L.W.); 2 State Key Laboratory of Animal Nutrition, Institute of Animal Sciences, Chinese Academy of Agricultural Sciences, Beijing 100193, China; cuishangjin@caas.cn

**Keywords:** porcine *Parvovirus*, VP2 protein, vaccine adjuvant, *N*-2-Hydroxypropyl trimethyl ammonium chloride chitosan, subunit vaccine

## Abstract

Porcine *parvovirus* (PPV) is the most important infectious agent causing infertility in pigs, which can be prevented by routine vaccination. Successful vaccination depends on the association with potent adjuvants that can enhance the immunogenicity of antigen and activate the immune system. Polysaccharide adjuvant has low toxicity and high safety, and they can enhance the humoral, cellular and mucosal immune responses. In the present study, we prepared the VP2 protein subunit vaccine against PPV (PPV/VP2/N-2-HACC) using water-soluble *N*-2-Hydroxypropyl trimethyl ammonium chloride chitosan (N-2-HACC) as the vaccine adjuvant, and the ability of the PPV/VP2/N-2-HACC to induce immune responses and protect sows from PPV infection was evaluated. In vivo immunization showed that the sows immunized with the PPV/VP2/N-2-HACC by intramuscular injection produced higher HI antibody levels and long-term immune protection compared with the other groups, while the subunit vaccine did not stimulate the proliferation of CD4+ and CD8+ T lymphocytes to trigger the secretion of higher levels of IL-2, IL-4, IFN-α, IFN-β, and IFN-γ, indicating that the PPV/VP2/N-2-HACC mainly induced humoral immunity rather than cellular immunity. PPV was not detected in the viscera of the sows immunized with the PPV/VP2/N-2-HACC, and the protective efficacy was 100%. Collectively, our findings suggested that the N-2-HACC was a potential candidate adjuvant, and the PPV/VP2/N-2-HACC had immense application value for the control of PPV.

## 1. Introduction

Porcine *parvovirus* (PPV) is the most important infectious agent causing infertility in pigs, leading to significant damage in sows and boars. PPV is mainly spread in the digestive tract and respiratory tract. PPV results in sterility, abortion, stillbirth, fetal mummy, and fetal malformation [1,2,3]. Additionally, PPV often combines with other pathogens to cause a mixed infection, directly resulting in sow breeding obstacles [4]. This disease causes huge economic losses to the pig breeding industry [5,6,7]. Nowadays, infections caused by PPV are very serious and have spread all over the world, and the positive antibody rate of swine exceeds 90%.

PPV is a small, non-enveloped and single-stranded DNA virus, which belongs to the genus *Parvovirus* and the family *Parvoviridae* [8]. The genome of PPV consists of two large open reading frames, which encode non-structural protein NS1 and several capsid proteins, such as VP1, VP2, and VP3 [9,10]. NS1 is necessary for viral DNA replication, and the VP2 protein is the major antigenic domain that can induce PPV-neutralizing antibodies and play a key role in the prevention and diagnosis of PPV [5]. Previous studies have demonstrated that the recombinantly expressed VP2 protein can self-assemble into virus-like particles, which have ideal immunogenicity and can stimulate the body to produce high antibody titers.

Classical inactivated PPV vaccine is available commercially and widely used to control PPV infections [11,12]. However, the safety and multiple immunizations with large doses are the major considerations of the inactivated vaccine, leading to the development of the PPV subunit vaccine. The adjuvant is an indispensable component of a subunit vaccines to improve the immunogenicity because it can induce stronger immune responses and reduce the dosage and production cost. A better adjuvant can not only enhance the immunogenicity of the vaccine and activate the immune system, such as cytotoxic T lymphocytes (CTLs) or helper T lymphocytes (THs), but also strengthen the humoral and/or cellular immune responses [13]. Natural polysaccharides are characterized by intrinsic immunomodulation, biocompatibility, biodegradability, low toxicity and safety, and a variety of natural polysaccharides have been proved to have better immune promoting effects and can enhance immune responses [14]. Among the natural polysaccharides, chitosan is a biopolymer of cationic polysaccharides with lots of biological properties, including low toxicity, good biocompatibility, and biodegradability [15,16], while the weak water solubility of chitosan greatly limits its application. One of the strategies to improve the solubility of chitosan is to modify the molecular structure by the addition of hydrophilic functional groups [17]. Our laboratory has synthesized the water-soluble *N*-2-Hydroxypropyl trimethyl ammonium chloride chitosan (N-2-HACC) [18], and the N-2-HACC has been used as the vaccine adjuvant and delivery system in the live-attenuated vaccine and DNA vaccine of Newcastle disease virus (NDV) [19,20]. In our previous studies, we found that the N-2-HACC nanoparticles can better carry the antigen into cells and enhance the immune responses, and the N-2-HACC can be used as a polysaccharide adjuvant to improve the immune level of the body. Many studies have shown that vaccine antigens encapsulated in or conjugated onto polymer-based microspheres/nanoparticles can stimulate the body to produce strong cellular, humoral and mucosal immune responses [21,22,23].

The effect of N-2-HACC in a non-nano state on vaccine immune enhancement, especially mucosal immune enhancement, is of great significance for the development of new chitosan derivative adjuvants. Therefore, we prepared the VP2 subunit vaccine against PPV (PPV/VP2/N-2-HACC) and aimed to explore the characteristics of PPV/VP2/N-2-HACC to evaluate the potential of N-2-HACC as a vaccine adjuvant of the subunit vaccine.

## 2. Materials and Methods

### 2.1. Cells, Virus, Commercial Vaccine, Adjuvant, and Animals

Recombinant *baculovirus* AcNPV-VP2, sf21 (*Spodoptera frugiperda*) cells, ST (Swine testis) cells, and PPV (PPV-H strain) were provided by the Research Team of Pig Digestive Tract Infectious Diseases of Harbin Veterinary Research Institute of the Chinese Academy of Agricultural Sciences (Harbin, China).

Commercial PPV inactivated vaccine (cp-99, 10^7.5^ TCID50/mL) was purchased from Shandong Binzhou Wohua Biological Engineering Co., Ltd. (Binzhou, Shandong Province, China), with the approval number of Veterinary Medicine (2009) 151721058. The water-soluble quaternized chitosan, *N*-2-Hydroxypropyl trimethyl ammonium chloride chitosan (N-2-HACC), was synthesized as previously described [18].

A total of 20 healthy sows (6~7 months old) and adult healthy boars were provided by the Harbin Veterinary Research Institute of the Chinese Academy of Agricultural Sciences (Harbin, China). Before the experiment, when the PPV antibody titers in the serum of sows was less than 2^6^ by haemagglutination inhibition (HI), the PPV antibody titers of all animals were confirmed to be negative in the study. All animal experiments were approved by the Animal Ethics Committee of Harbin Veterinary Research Institute of the Chinese Academy of Agricultural Science and the Animal Ethics Committee of Heilongjiang Province (SYXK (H) 2006-032). All animals received humane care in compliance with good animal practice according to the animal ethics procedures and guidelines of China.

### 2.2. Expression and Purification of PPV VP2 Protein

Expression and purification of the PPV VP2 protein were carried out as previously described [24]. Briefly, the PPV VP2 protein was expressed in an insect-*baculovirus* cell system and purified using Ni-NTA affinity column chromatography. The purified VP2 protein was separated by SDS-PAGE and then electro-transferred onto nitrocellulose membrane for Western blotting analysis. The membranes were blocked with 5% skim milk in TBST buffer (50 mmol/L Tris-HCl, 150 mmol/L NaCl, 0.05% Tween 20, pH 7.5) at 4 °C overnight; Subsequently, the membranes were incubated with swine anti-PPV serum obtained from the Harbin Veterinary Research Institute at a dilution of 1:1000 with shaking overnight at 4 °C. The membranes were then washed three times with TBST buffer, each time for 5 min, followed by incubation with horseradish peroxidase-conjugated rabbit anti-pig IgG antibody (Sigma, St. Louis, MO, USA) for 1 h, and then the membranes were washed with TBST buffer three times. The immunoreactive bands were visualized by a Licor ODYSSEY near-infrared fluorescent scanning imaging system (Licor, Lincoln, NE, USA).

### 2.3. Preparation of the PPV/VP2/N-2-HACC

Hemagglutination (HA) titer of the VP2 protein was tested by HA assay [25]. The VP2 protein solution was mixed with the N-2-HACC solution at a ratio of 0.5% (*v*/*v*) and then emulsified with mineral oil for 30 min at a speed of 8000 r/min. The subunit vaccine was abbreviated as PPV/VP2/N-2-HACC. The HA titer of the VP2 protein in the PPV/VP2/N-2-HACC was 2^8^. 

### 2.4. Sterility Assay of the PPV/VP2/N-2-HACC

To determine the sterility of the PPV/VP2/N-2-HACC, the extraneous bacteria or mold were tested as previously described [26]. Briefly, 0.2 mL of the PPV/VP2-N-2-HACC solution was inoculated onto the thioglycolate medium (T.G), tyropeptone agar medium (G.A), and glucose peptone medium (G.P) under the aseptic condition. Subsequently, the plates were incubated at 37 °C and 28 °C for 3–5 days to determine the presence of bacteria or mold. Meanwhile, the endotoxins in the PPV/VP2/N-2-HACC were detected by the Limulus test.

### 2.5. Safety and Storage Stability of the PPV/VP2/N-2-HACC

To assess the safety of PPV/VP2/N-2-HACC, in vivo cytotoxicity assays were carried out on five 4-month-old healthy sows. Each sow was intramuscularly injected with 20 mL of the PPV/VP2/N-2-HACC at multiple sites. The abnormal changes in the administered sow, such as injection site, feeding, drinking, mental state, body weight, and clinical symptoms, were continuously monitored and recorded for 14 days. 

To investigate the storage stability of PPV/VP2/N-2-HACC, the subunit vaccine was stored at room temperature, 4 °C, −20 °C, and 37 °C for 28 days, respectively. The morphology, particle size, viscosity, and stability of the PPV/VP2/N-2-HACC were assessed and recorded.

### 2.6. Vaccination

A total of 20 sows were randomly and evenly divided into four groups, and the animals were housed separately. Sows in Group 1 were immunized with 2 mL PPV/VP2/N-2-HACC; sows in Group 2 were immunized with 2 mL commercial PPV inactivated vaccine; and sows in Groups 3 and 4 were immunized with 2 mL N-2-HACC or PBS, respectively. The sows were immunized by intramuscular injection in the neck, and the booster injection was given at an interval of 2 weeks. After the second immunization, sows of each group were mated with serum PPV-negative adult healthy boars, and the fetuses in the uterus of sows were observed, including sterility, abortion, stillbirth, fetal mummy, and fetal malformation. Blood samples were aseptically collected from the anterior aortic vein of sows on 0, 7, 14, 21, 28, 35, 42, and 56 days after the first immunization, respectively. Serum HI antibody titers were measured by a micro-method [27], and the contents of cytokines (IFN-α, IFN-β, IFN-γ, IL-2, and IL-4) in serum were measured by corresponding ELISA kits (Invitrogen, Carlsbad, CA, USA).

To investigate the cellular immune response induced by the PPV/VP2/N-2-HACC, the blood samples were aseptically collected from the anterior aortic vein on 28, 42, 56, 70, and 84 days after the first immunization, respectively. The contents of CD4+ and CD8+ T lymphocytes were measured as follows. Lymphocytes were incubated with FITC-conjugated mouse anti-pig CD4+ antibody and phycoerythrin (PE)-conjugated mouse anti-pig CD8+ antibody. After incubation, the cells were washed with cold PBS three times and then suspended in PBS. The CD4+ and CD8+ T lymphocytes were sorted by FACSAria flow Cytometer (BD Biosciences, San Diego, CA, USA).

### 2.7. Protective Efficacy

The ability of PPV/VP2/N-2-HACC to protect sows against the infection of PPV-H strain (10^6.0^ TCID50/mL) was determined. When the level of HA antibody in serum of every immunization group reached 7.0 log2 after the first immunization, five sows from each group were intramuscularly injected with 2.0 mL of the highly virulent PPV-H for challenge studies. Any abnormal changes, such as feeding, water drinking, mental state, body weight, clinical symptoms and mortality of these sows were continuously observed and recorded for 28 days. The blood was collected for the detection of serum HI antibody level as well as the contents of CD4+ and CD8+ T lymphocytes at 1, 2, 3, and 4 weeks after the challenge. At the 4th week after the challenge, sows were euthanized, and the fetuses in the uterus of sows were observed, including the total number of fetuses, number of diseased fetuses, stillbirth, fetal mummy, and fetal malformation. Meanwhile, the hearts, lungs, livers, spleens, and kidneys from sows were collected for histological staining assay. The viral DNAs in the heart, lung, liver, and spleen (200 mg) was extracted with the DNA Extraction Kit (Biotech Corporation, Beijing, China) and stored at −20 °C. The quantitative real-time polymerase chain reaction (qRT-PCR) was used to detect the content of PPV in sows after the challenge and assess the immune effect [28].

Specific primers for PPV (forward primer: 5′-AAATGTACCTGATTGGTCCG-3′, reverse primer: 5′-TTGGTGAAAGTTGGTGTTGTT-3′) were designed based on a partial fragment of NS1 using Primer-BLAST software. The probe (5′-CAGCGGACAACAACTACGCAGCA-BHQ1-3′) was synthesized by Shanghai Invitrogen Trade (Shanghai, China). The qRT-PCR was performed in a 20 μL reaction system consisting of 10 μL of Premix Ex Taq Probe qRT-PCR (TaKaRa, Kusatsu-shi, Japan), 0.5 μL of PPV forward primer (10 nmol/mL), 0.5 μL of PPV reverse primer (10 nmol/mL), 1 μL of probe (10 nmol/mL), 1 μL of genomic DNA, and 7 μL of *DNase*/*RNase*-free deionized water. Briefly, after an initial denaturation step at 95 °C for 5 min, the amplifications were carried out with 45 cycles at a melting temperature of 95 °C for 10 s, an annealing temperature of 58 °C for 30 s, and an extension temperature of 72 °C for 20 s. Fluorescent signals were detected at the end of each cycle. The cycle threshold (CT) value was defined as the cycle number, at which the amplification curve reached the threshold. Samples with a CT value above the cutoff value were retested and considered positive if they remained so.

### 2.8. Statistical Analysis

Data were presented as the mean ± standard deviation (SD). Duncan’s multiple range test was used to determine the differences among groups with SPSS 19.0 (IBM, New York, NY, USA). *p* < 0.05 was considered statistically significant.

## 3. Results

### 3.1. Expression of the PPV VP2 Protein In Vitro

**Figure 1:**
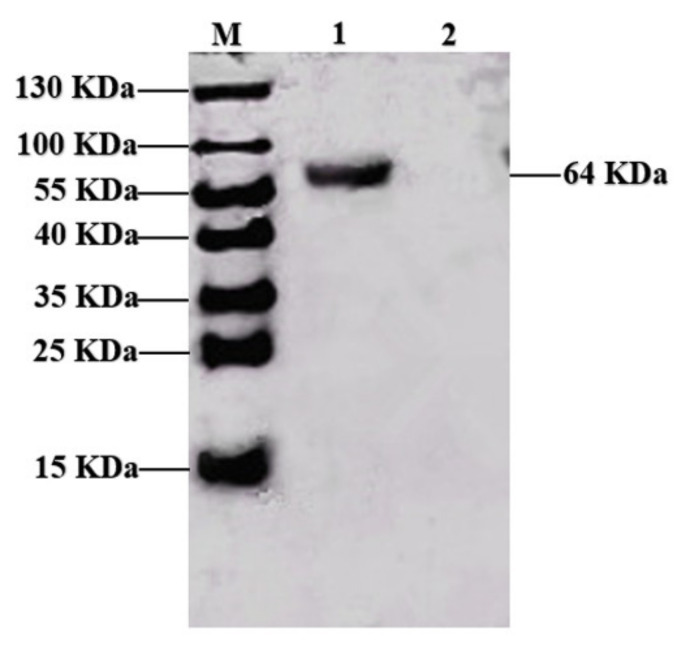
Western blotting analysis of the PPV VP2 expressed in sf21 cells. M: Protein ladder; 1: Precipitation of cells (the purified VP2); 2: Supernatant of the cell culture. To confirm the PPV VP2 expression, the purified VP2 was subjected to Western blotting analysis. The primary antibody is PPV-positive pig serum, and the secondary antibody is horseradish peroxidase-conjugated rabbit anti-pig IgG antibody.

Figure 1 shows that the PPV VP2 protein was purified. To confirm the expression of the VP2 protein, the purified VP2 protein was analyzed by Western blotting analysis using PPV-positive pig serum, and the polyclonal antibodies recognized VP2 protein with a molecular weight of 64 KDa (Figure 1), and the densitometry readings of target bands were 10,717 using the Image J software (NIH, Bethesda, MD, USA).

### 3.2. Sterility of the PPV/VP2/N-2-HACC

The prepared PPV/VP2/N-2-HACC was milky white liquid and easily detached from the bottle wall by gently shaking, and the lower layer was slightly reddish after standing. There was no growth of bacteria and mold on the T.G, G.A, and G.P medium containing the PPV/VP2/N-2-HACC. The content of endotoxin in the PPV/VP2/N-2-HACC was less than 0.125 EU/mL, which was following the stipulations of the third volume (2010 edition) of Veterinary Pharmacopoeia of the People’s Republic of China (less than 5.0 EU/mL). These results showed that there was no pathogenic microbial contamination in the PPV/VP2/N-2-HACC, and the subunit vaccine could be used safely.

### 3.3. Safety of the PPV/VP2/N-2-HACC

In vivo toxicity results showed that the feeding, drinking, mental state, and body weight were normal in the sows immunized with the high doses of PPV/VP2/N-2-HACC, and the morbidity and mortality of sows were 0%. Furthermore, there were no inflammation, redness, and other abnormal phenomena at the injection site. These results indicated that the high doses of the PPV/VP2/N-2-HACC were safe.

### 3.4. Storage Stability of the PPV/VP2/N-2-HACC

To evaluate the storage stability of the PPV/VP2/N-2-HACC, the subunit vaccine was placed at room temperature, 4 °C, −20 °C, and 37 °C for 28 days. The results showed that no significant changes in the morphology and particle size of the PPV/VP2/N-2-HACC were observed after the PPV/VP2/N-2-HACC was placed for 28 days at room temperature, 4 °C, and −20 °C. However, it was slightly stratified after being stored at 37 °C for 28 days. In addition, it took 6.0 s to naturally flow out 400 μL of the PPV/VP2/N-2-HACC stored at different temperatures for 28 days, showing that the viscosity of the subunit vaccine was satisfactory. When the PPV/VP2/N-2-HACC stored at different temperatures for 28 days was centrifuged at 3000 r/min for 20 min, it was still milky white, and there was no delamination and demulsification. These results indicated that the subunit vaccine had good storage stability and could be placed for a long period even at room temperature. The immunization power of the vaccine after being placed at room temperature, 4 °C, −20 °C, and 37 °C for 28 days will be evaluated in further study. 

### 3.5. PPV/VP2/N-2-HACC Promotes Antibody Production

**Figure 2:**
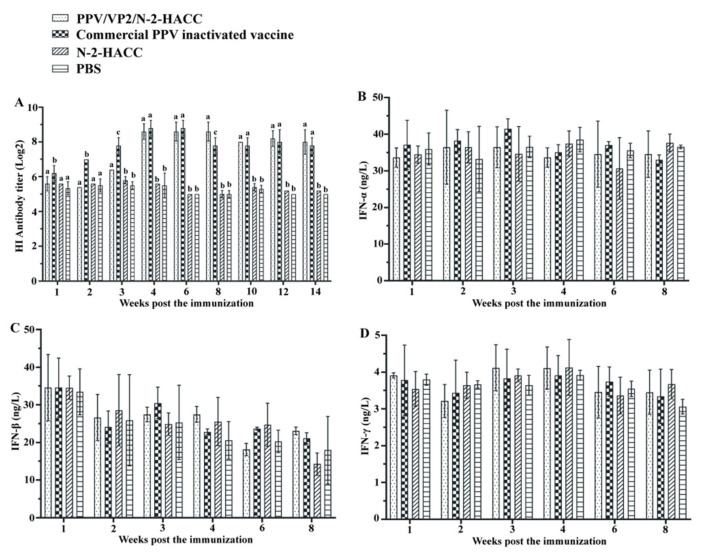
PPV-specific antibody titers (**A**) and the levels of IFN-α (**B**), IFN-β (**C**), and IFN-γ (**D**) in serum from the sows immunized with the PPV/VP2/N-2-HACC, commercial PPV inactivated vaccine, N-2-HACC and PBS via intramuscular injection. Values represent mean ± S.D. (*n* = 5). Note: a, b, c: Values within the same column with the different lower case letters (a–c) in the superscript indicate statistically significant differences (*p* < 0.05).

Figure 2A shows that no antibody was detected in the N-2-HACC and PBS groups. PPV/VP2 specific antibody was detected at 14 days and 21 days post the immunization in the commercial PPV inactivated vaccine and PPV/VP2/N-2-HACC groups, respectively. From 70 days after the immunization, the antibody titers in serum from the sows immunized with the PPV/VP2/N-2-HACC were higher compared with those immunized with the commercial PPV inactivated vaccine, while the differences in both groups were not significant (*p* > 0.05), and the antibody titer in the PPV/VP2-N-2-HACC group remained at a high level for more than 14 weeks, suggesting that the PPV/VP2/N-2-HACC induced better humoral immune response compared with the commercial PPV inactivated vaccine. Furthermore, the subunit vaccine induced long-lasting antibody levels compared with the commercial PPV inactivated vaccine. Additionally, HI antibody titers in serum from sows immunized with the PPV/VP2/N-2-HACC and commercial PPV inactivated vaccine were detected at four weeks after the immunization, and the immune protection against PPV infection was induced in the sows in both groups.

### 3.6. PPV/VP2/N-2-HACC Does Not Enhance Cellular Immune Response

**Figure 3:**
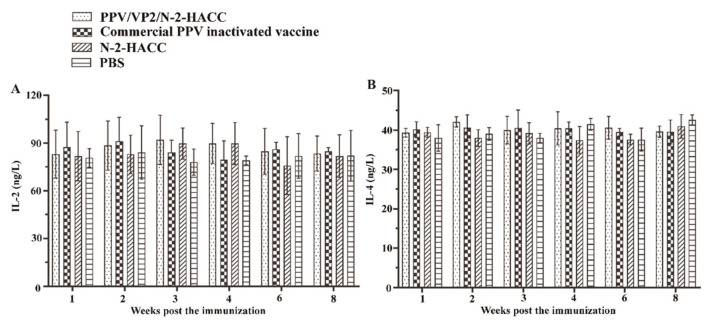
The levels of IL-2 (**A**) and IL-4 (**B**) in serum from the sows immunized with the PPV/VP2/N-2-HACC, commercial PPV inactivated vaccine, N-2-HACC, and PBS via intramuscular injection. Values represent mean ± S.D. (*n* = 5).

To evaluate the cellular immune response induced by the PPV/VP2/N-2-HACC, we investigated the levels of IFN-α, IFN-β, IFN-γ, IL-2, and IL-4, as well as the content changes of CD4+ and CD8+ T lymphocytes, in each immunized group. From first to eighth week post the immunization, the levels of IFN-α (Figure 2B), IFN-β (Figure 2C), IFN-γ (Figure 2D), IL-2 (Figure 3A), and IL-4 (Figure 3B) in the PPV/VP2/N-2-HACC and commercial PPV inactivated vaccine groups had no significant difference compared with those of the control group (*p* > 0.05). 

**Figure 4:**
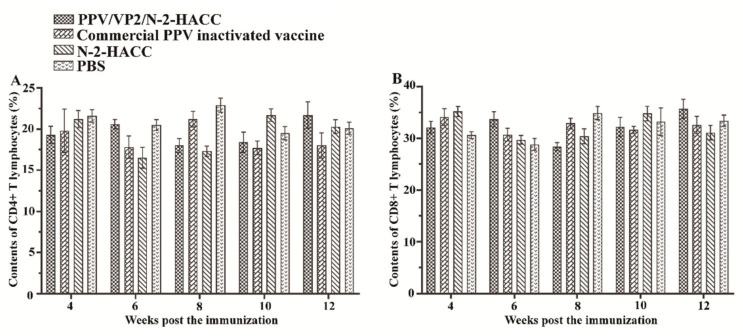
The contents of CD4+ (**A**) and CD8+ (**B**) T lymphocytes in the blood from the sows immunized with the PPV/VP2/N-2-HACC, commercial PPV inactivated vaccine, N-2-HACC, and PBS via intramuscular injection. Values represent mean ± S.D. (*n* = 5).

Figure 4 shows that the contents of CD4+ and CD8+ T lymphocytes from the 4th to 12th week post the immunization were not significantly different between the vaccine-immunized groups and control groups (*p* > 0.05). These results indicated that both the PPV/VP2/N-2-HACC and commercial PPV inactivated vaccine did not induce the cellular immune response.

### 3.7. PPV/VP2/N-2-HACC Protects Pigs from PPV Infection

**Figure 5:**
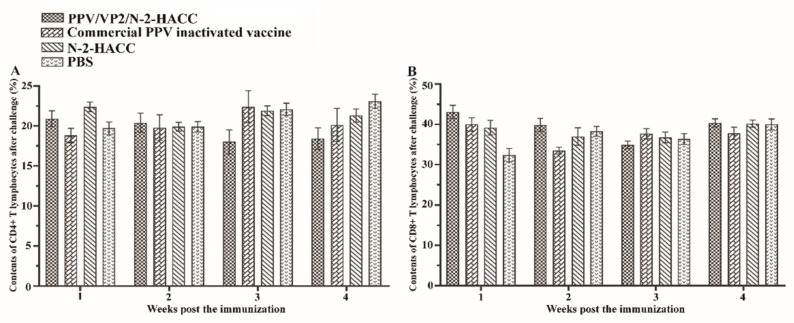
Contents of CD4+ (**A**) and CD8+ (**B**) T lymphocytes in the peripheral blood after challenge. Values represent mean ± S.D. (*n* = 5).

The PPV/VP2 specific antibody was measured by ELISA assay after the challenge (Table 1). HI antibody titers in the N-2-HACC and PBS groups were all at a low level. The levels of HI antibody in the PPV/VP2/N-2-HACC group were significantly higher compared with the commercial PPV inactivated vaccine group in the first and third week after challenge (*p* < 0.05), while HI antibody between the PPV/VP2/N-2-HACC group and commercial PPV inactivated vaccine group had no significant difference in the second and fourth week after challenge. The contents of CD4+ (Figure 5A) and CD8+ T lymphocytes (Figure 5B) in the peripheral blood had no significant changes after the challenge between the PPV/VP2/N-2-HACC and commercial PPV inactivated vaccine groups (*p* > 0.05).

The ability of PPV/VP2/N-2-HACC to eliminate PPV infection was assessed after the challenge with PPV. The high-titer viruses from different organs were detected, and no virus was detected in the organs of all the sows immunized with the PPV/VP2/N-2-HACC and commercial PPV inactivated vaccine at 28 days after the immunization. 

Five sows immunized with the PPV/VP2/N-2-HACC and commercial PPV inactivated vaccine displayed no disease symptoms and survived until 28 days after the challenge. The sows in the N-2-HACC and PBS groups showed clinical symptoms, while the sows in the PPV/VP2/N-2-HACC and commercial PPV inactivated vaccine groups did not show clinical symptoms (Table 2). The protective efficacy was 100% for the sows immunized with the PPV/VP2/N-2-HACC and commercial PPV inactivated vaccine. The fetuses in the uterus of sows immunized with the commercial PPV inactivated vaccine were normal, while the fetuses of the N-2-HACC and PBS groups showed various degrees of fetal mummification (Table 2). Because the sows immunized with the PPV/VP2/N-2-HACC were not successfully mated, the results of fetuses from sows immunized with the PPV/VP2/N-2-HACC were not shown in Table 2.

**Figure 6:**
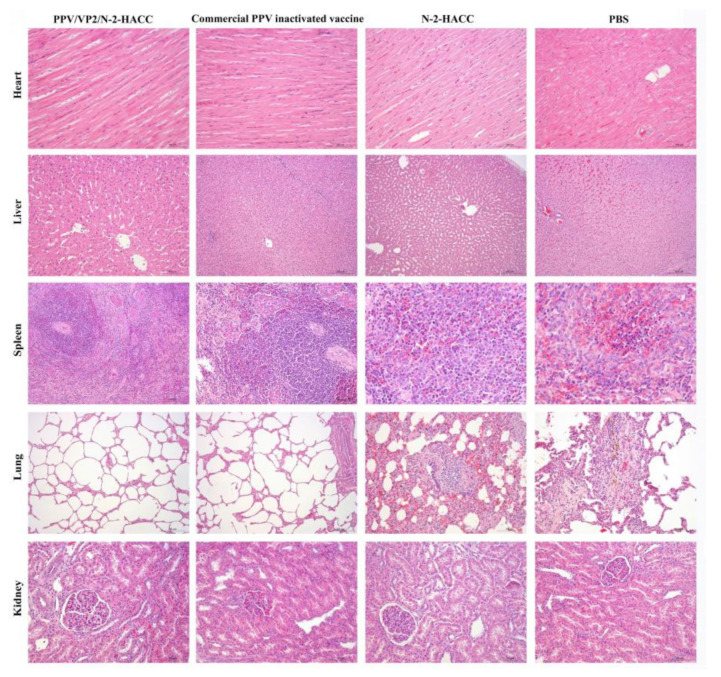
Histopathological analyses of heart, liver, spleen, lung, and kidney from the immunized sows at 28 days after the challenge with the highly virulent PPV.

Histologically, the kidney surface of sows in the PBS and N-2-HACC groups had a large number of bleeding spots, the surfaces of the liver, spleen, and heart had dark red hemorrhages, and the pathological sections showed local inflammation. There were a large number of red blood cells and white blood cells in alveoli from sows in the PBS and N-2-HACC groups, the capillaries in the alveolar walls were congested with many red blood cells, and there was hemosiderosis. However, the heart, liver, spleen, lung, and kidney from sows immunized with PPV/VP2/N-2-HACC and commercial PPV inactivated vaccine significantly eliminated the viral infection during the challenge, and no obvious pathological changes were found (Figure 6). Additionally, organs obtained from sows immunized with the PPV/VP2/N-2-HACC showed less pathological changes compared with the commercial PPV inactivated vaccine group (Figure 6).

## 4. Discussion

PPV causes reproductive failure in pregnant sows, and vaccine immunization is considered to be the most valuable and valid way to prevent PPV. Current vaccines have some flaws [29,30,31], and it is necessary to develop a novel adjuvant to improve the effectiveness of the PPV vaccine. Chitosan nanoparticle delivery systems have been used for encapsulation of antigen, which enhances antibody responses compared with the traditional adjuvant. We have previously evaluated the potential of chitosan nanoparticles as an adjuvant in the NDV vaccine and avian influenza virus (AIV) vaccine in chickens. However, the water solubility of chitosan is weak, which limits its application as an adjuvant [32]. Our previous study has proved that the water-soluble N-2-HACC can serve as a safe vaccine adjuvant and delivery carrier for the NDV vaccine [33]. Therefore, in the present study, we investigated the potential of the water-soluble N-2-HACC as an adjuvant to enhance protective immune responses of the VP2 subunit vaccine in sows. In vivo safety results showed that the sows’ diets and spirits were normal and there were no signs of morbidity after intramuscular injecting 10 times dose of the PPV/VP2/N-2-HACC, and the PPV/VP2-N-2-HACC generated significantly higher systemic antibody responses and protected sows against PPV infection. Therefore, the PPV/VP2-N-2-HACC could be a potential vaccine candidate.

The immune system is diverse with the capability of responding to all types of infectious organisms [34]. Humoral immunity plays an important role in the immune system. Higher antibody titers and longer duration play an important role in preventing and defending against epidemic diseases. Nanocarrier-based delivery systems can provide a suitable route of administration and induce robust humoral immune responses. In the present study, at the first to third week after the immunization, the HI antibody levels of commercial PPV inactivated vaccine were higher compared with the PPV/VP2-N-2-HACC (*p* < 0.05). However, the levels of HI antibody in the PPV/VP2/N-2-HACC group were increased rapidly at 28 days after the immunization, and then the upward trend was relatively flat until 56 days after the immunization. From 70 days after the immunization, the serum HI antibody levels of PPV/VP2-N-2-HACC were higher compared with the commercial PPV inactivated vaccine, while the differences in both groups were not significant (*p* > 0.05). The antibody titer in the PPV/VP2-N-2-HACC group remained at a high level for more than 14 weeks. These results showed that the immunity linked to the administration of commercial PPV inactivated vaccine started earlier, the PPV/VP2-N-2-HACC had better results in the long period, and the PPV/VP2/N-2-HACC enhanced the humoral immunity.

The cytokines secreted by helper T cells can be divided into Th1-related cytokines (IFN-γ and IL-2) and Th2-related cytokines (IL-4 and IL-6) [35]. Two types of cytokines are mutually restricted in the body to maintain the body’s immune balance [36,37]. IFN-γ mediates cellular immunity by promoting the proliferation and differentiation of T cells, and it is indispensable for the activation of macrophages that ingest and kill intracellular pathogens. IFN-γ and IL-12 promote IgG2a production (Th1 type) [38]. IL-2 is an important component for the activation and differentiation of T lymphocytes. In addition, it can promote the secretion of immunoglobulins produced by B cells and induce the differentiation and proliferation of natural killer cells [39]. IL-4 plays an important role in Th2 differentiation and strongly induces the secretion of IL-5, IL-9, and IL-10 [40]. Besides, IFN-α and IFN-β are important members of the interferon family and belong to type I interferon [41]. IFN-α has cytotoxic activity, and IFN-β is secreted by immune cells, both playing important roles in cellular immunity [42]. In the present study, the contents of IL-2, IL-4, IFN-α, IFN-β, and IFN-γ were detected in the serum of sows immunized with the PPV/VP2/N-2-HACC and commercial PPV inactivated vaccines, while there was no significant difference among these groups. It indicated that the PPV/VP2/N-2-HACC failed to induce Th1 and Th2 immune responses. T-helper lymphocytes (CD4+) and T-cytotoxic lymphocytes (CD8+) are key links in the immune regulation of organisms. CD4+ T lymphocytes can promote a cell-mediated immune response and induce the maturation of Ts and Th cells. CD8+ T lymphocytes can recognize and kill the infected cells in the host [43]. Cellular immunity plays a role in clearing the long-term viral infection. Therefore, it may be relevant for long-term PPV infection [44]. In the present study, the contents of CD4+ and CD8+ T lymphocytes in the serum of sows immunized with the PPV/VP2/N-2-HACC and commercial PPV inactivated vaccine did not change after immunization and challenge. The changes of CD4+ and CD8+ T lymphocytes were consistent with the cytokine test results. Therefore, the PPV/VP2/N-2-HACC induced good humoral immunity in the body, while it did not induce cellular immunity.

Numerous derivatives of chitosan with varying chemical and physical properties have been synthesized in recent years. In the present study, our results showed that the prepared PPV/VP2/N-2-HACC had good storage stability, no pathogenic microbial contamination, safety with high doses, and fewer pathological changes after being challenged with PPV-H strain. Moreover, the adjuvanticity of water-soluble chitosan was evaluated, and the result showed that the water-soluble chitosan significantly improved the protective effec of the PPV subunit vaccine against PPV infection. Our findings indicated that the N-2-HACC was a potential candidate adjuvant and could be highly useful for the rational development of improved vaccines, suggesting the possibility of the PPV/VP2/N-2-HACC to be a novel efficient PPV vaccine. 

## 5. Conclusions

These results indicated that the PPV/VP2/N-2-HACC nano subunit vaccine was safe and had good storage stability and could be placed for a long period even at the room temperature. While results of the immunization showed that the immunity linked to administration of commercial PPV inactivated vaccine started earlier, the PPV/VP2-N-2-HACC had better results in the long period.

## Figures and Tables

**Table 1:** HI antibody titers in serum after challenge (unit: Log2).

Groups	Weeks after Challenge			
	1	2	3	4
PPV/VP2/N-2-HACC	9.5 ± 0.7 ^a^	10.5 ± 0.71 ^a^	10.0 ± 0.0 ^a^	10.0 ± 1.41 ^a^
Commercial PPV inactivated vaccine	8.0 ± 0.0 ^b^	10.5 ± 0.71 ^a^	11 ± 1.41 ^b^	10.0 ± 0.0 ^a^
N-2-HACC	7.0 ± 0.0 ^c^	7.5 ± 0.71 ^b^	7.0 ± 0.0 ^c^	6.5 ± 0.71 ^b^
PBS	7.5 ± 0.71 ^c^	7.0 ± 1.41 ^b^	7.5 ± 0.71 ^c^	7.0 ± 0.0 ^b^

Note: Values within the same column with the different lower case letters (a–c) in the superscript indicate statistically significant differences (*p* < 0.05).

**Table 2:** Protective efficacy of immunized sows after challenged with PPV-H strain.

Groups	The Number of Sows with Clinical Symptoms/Total Sows	The Number of Diseased Fetuses/Total Fetuses
PPV/VP2/N-2-HACC	0/5	—/—
Commercial PPV inactivated vaccine	0/5	0/5
N-2-HACC	5/5	5/5
PBS	5/5	3/3

## Data Availability

Not applicable.
